# A New Spatial Registration Algorithm of Aerial Moving Platform to Sea Target Tracking

**DOI:** 10.3390/s23136112

**Published:** 2023-07-03

**Authors:** Qiuyang Dai, Faxing Lu

**Affiliations:** College of Weaponry Engineering, Naval University of Engineering, Wuhan 430033, China

**Keywords:** target tracking, spatial registration, nonlinear filtering, aerial moving platform, interactive multi-model

## Abstract

Spatial registration is the primary challenge affecting target tracking accuracy, especially for the aerial moving platform and sea target tracking. In this environment, it is important to account for both the errors in sensor observations and the variations in platform attitude. In order to solve the problem of complex types of errors in the tracking of sea targets by aerial moving platforms, a new spatial registration algorithm is proposed. Through separating and analyzing observation data, the influence of sensor observation error and attitude error on observation data is obtained, and a systematic error consistency matrix is established. Based on observation information from multiple platforms, accurate tracking of sea targets can be accomplished without estimating systematic error. In order to verify the effectiveness of the algorithm, we carried out simulation experiments and practical experiments on the lake, which showed that the new algorithm was more efficient than traditional algorithms.

## 1. Introduction

Sea target tracking usually deals with uncertainty problems, which mainly originate from dynamic uncertainty caused by the non-collaborative nature of the target and measurement uncertainty caused by measurement noise. To solve the two key problems of target dynamic uncertainty and target measurement uncertainty, a large number of theories and techniques have been generated for the study of target tracking.

In the 1960s, Kalman et al. [1] proposed a Kalman filter, which has the advantages of small computation and a simple process, and is the optimal estimation in the sense of the minimum mean square error under linear conditions, so it has been widely used. However, Kalman filtering can only deal with linear models. Scholars have carried out much research work in order to deal with nonlinear model problems. The first one is the Extended Kalman Filter (EKF) [2]. However, this method has only first-order estimation accuracy. In order to improve the estimation accuracy, the Unscented Kalman Filter (UKF) [3] with second-order estimation accuracy was subsequently proposed. However, this filtering method suffers from low filtering accuracy in high-dimensional systematics. To overcome this difficulty [4], researchers proposed the Cubature Kalman Filter (CKF). The above filtering methods all belong to the category of point estimators. There is another important class of methods for nonlinear state estimation, called probability density estimators [5,6,7]. Particle filtering is a typical representative, which can solve the state estimation problem under the noise of a non-Gaussian distribution [8]. For systematics with a high degree of nonlinearity, probability density estimators can be used to obtain state estimates with higher accuracy than point estimators, but probability density estimators are computationally intensive and often difficult to complete in real-time. With the spurt of artificial intelligence in recent years, many scholars have tried to apply deep learning to target tracking and the design of filters. Ref. [9] obtains filters with a more uniform form and stronger performance by constructing a generalized delayed feedback structure.

The above methods are more effective in solving tracking problems for a single model, but the accuracy decreases when estimating the state of a target with multiple motion patterns. However, in practical scenarios, targets inevitably have certain maneuvering characteristics, and their state models tend to change over time when the targets are maneuvering. To solve the tracking problem of maneuvering targets [10], an Interacting Multiple Model (IMM) algorithms was proposed, which has been proven to be one of the most cost-effective schemes for hybrid systematic estimation. The current combination of IMM and filtering algorithm [11,12,13] shows good results in single-sensor maneuvering target tracking, but still suffers from poor processing accuracy of observed information and low computational efficiency in multi-sensor cooperative target tracking.

The sensors carried by the aerial moving platform will inevitably generate measurement errors due to their own performance indicators, systematic regimes, zero-value correction residuals, interference, and noise. Such errors include random errors and systematic errors. Random error can be reduced by filtering techniques, but systematic error is an inherent error that is difficult to eliminate by filtering methods [14,15,16,17]. Even if the systematic error of the sensor is eliminated by manual calibration before use, the platform attitude angle error is coupled with the sensor observation error during the flight of the aerial moving platform, and the systematic error of the sensor will be regenerated over time. It is a dynamic process of change. This systematic error seriously affects the positioning accuracy of the sensor relative to the target and further affects the fusion effect of multi-sensor cooperative target tracking. Therefore, it is necessary to eliminate and align systematic errors. In addition, error alignment technology is also a fundamental support technology for the recent rapid development of mosaic warfare and decision-centered warfare, which are also hot issues in the military field.

Scholars have proposed a variety of spatial registration methods for systematic spatial registration, such as the real-time quality control method [18], the least squares method [19], the generalized least squares method [20], the exact maximum likelihood method [21], and so on. The emergence of these traditional algorithms has promoted the progress of spatial registration techniques. However, these algorithms estimate the systematic error in an offline manner and cannot handle the time-varying systematic error. In response to the problem of the poor real-time performance of traditional algorithms, online real-time abatement algorithms have been proposed, mainly including the extended dimensional Kalman algorithm, the decoupled filtering algorithm, and the exact algorithm. Okello et al. [22] proposed a Bayesian framework for error ablation and fusion estimation for sensors with constant or slowly varying systematic errors. Wang et al. [23] designed a two-stage Kalman filtering algorithm for online estimation of navigation systematic error and sensor systematic error to achieve error alignment for multiple platforms at sea. To address the shortcomings of the scattering-prone filtering of the expanded-dimensional Kalman algorithm, Song et al. [24] introduced the UKF algorithm into the error alignment problem to realize joint estimation of systematic error and target state.

The above algorithms only consider the sensor observation systematic error, which can solve the error alignment problem of fixed platforms well. Aerial moving platforms such as UAVs, unlike fixed platforms, have an attitude angle error, and this error couples to sensor observation error. The effect of this error is not only nonlinear, but also related to the position of the target. It exhibits time-varying sensor measurement systematic error, causing difficulties in estimation. Ref. [25] divides the estimation of sensor observation systematic error and attitude angle error into two steps. Firstly, the attitude angle is set to zero and the Kalman filter is used to estimate the sensor observation systematic error; then the sensor observation is corrected by the obtained sensor systematic error and then the attitude angle error is estimated by the Kalman filter, but this method does not consider the coupling effect of the attitude angle error on the sensor error. The above research content uses the relative attitude angle systematic error and the relative sensor systematic error between two sensors as the state vector, so only the relative systematic error can be estimated.

For the systematic spatial registration problem of dynamic platform sensors, there are generally two systematic error estimation models. The first one is a fully expanded three-dimensional model. It forms a state vector including the systematic error of the sensor and the attitude angle systematic error to estimate. Cui et al. [26] propose an improved Exact Method (EX) algorithm and then extend the improved EX algorithm to effectively estimate the attitude angle error and the sensor systematic error to solve the systematic error alignment problem of maneuvering radar. However, this algorithm is greatly influenced by the measurement points and is not very stable. Ref. [27] extends the great likelihood spatial registration algorithm based on a fixed platform to the maneuvering platform. It further considers the problem of the existence of attitude angle systematic error in the maneuvering platform and the effective estimation of sensor systematic error, attitude angle systematic error, and target state. However, this algorithm is an offline estimation method and cannot solve the time-varying sensor systematic error abatement problem. Wang et al. [28] construct a pseudo-measurement model of the ship attitude angle systematic error and the sensor observation systematic error based on the ECEF method (Earth Centered Earth Fixed, ECEF), and use the Kalman filtering algorithm to realize the real-time online estimation of the alignment error.

The second model is a decoupled, fully expanded dimensional model that equates the attitude angle systematic error to the sensor systematic error. Ref. [29] establishes the systematic error state equation and measurement equation based on the sensor equivalent measurement error caused by the attitude angle error. It estimates the attitude angle systematic error and sensor goniometric systematic error of the moving platform using the UKF algorithm and the sensor goniometric systematic error using the generalized least squares method. Ref. [30] uses the known position information of the cooperative target to equate the attitude angle error of the moving platform as a part of the sensor observation systematic error and establishes a decoupling model of the sensor systematic error to realize the real-time estimation of the attitude angle systematic error and the sensor systematic error. Wang et al. [31] use a linearization method to give an expression for the equivalent measurement error caused by the attitude angle systematic error. They discard the pitch angle systematic error and the cross-roll angle systematic error when selecting the state vector while treating the deviation of the sensor azimuth angle systematic error from the yaw angle systematic error as a variable to construct the state equation and the measurement equation, and then use the Kalman filtering algorithm to estimate the systematic error.

Therefore, in order to obtain the expected result of the aerial moving platform for tracking the target on the sea, the following two problems need to be solved: firstly, the attitude angle systematic error and random error of the aerial moving platform and the sensor systematic error and the random error should be reduced; secondly, a suitable tracking algorithm should be used for fast and accurate processing of the obtained data.

In order to solve the above problems, we propose a new tracking algorithm for sea targets based on the sensor spatial registration of an aerial moving platform. Firstly, the observation error of the aerial moving platform for sea target detection is modeled and analyzed, and the influence of sensor error and attitude angle error on the observation data is derived. Then, we use two aerial moving platforms to detect sea targets and complete the reduction of sensor systematic error and attitude angle systematic error of aerial moving platforms. Finally, the IMM algorithm and Kalman filtering algorithm are combined to complete the tracking of the sea target. Through simulation experiments, on-lake experiments, and comparative analysis, the feasibility and effectiveness of the method are verified.

The organization of the rest of the paper is as follows: Section 2 gives a brief overview of the detection model of an aerial moving platform and essential position alignment. Section 3 describes the proposed new spatial registration algorithm, and Section 4 details the simulation experiment and the practical experiment. Finally, a few concluding remarks are presented in Section 5.

## 2. Detection Model and Position Alignment

### 2.1. Detection Model of Aerial Moving Platform

First, we describe the detection model of the aerial moving platform to the sea target. At a certain moment, the aerial moving platform acquires high-precision geographic coordinates $X_{e}={\left[\begin{array}{ccc} L & B & H \end{array}\right]}^{T}$, where *L* represents longitude, *B* represents latitude, and H represents geographic height. The measurement result of the platform sensor is affected by the observation error B as $Z_{um}={\left[\begin{array}{ccc} r_{um} & b_{um} & e_{um} \end{array}\right]}^{T}$. Assuming the true position of the sea target in the unstable carrier coordinate systematic of the platform is $X_{ut}={\left[\begin{array}{ccc} x_{ut} & y_{ut} & z_{ut} \end{array}\right]}^{T}$, the measurement equation of the aerial moving platform is

(1) $$Z_{um}=h_{rtc}(X_{ut})+B_{s}+B_{w}$$

where $Z_{um}={\left[\begin{array}{ccc} r_{um} & b_{um} & e_{um} \end{array}\right]}^{T}$ denotes measured value in unstable carrier coordinate system, $X_{ut}={\left[\begin{array}{ccc} x_{ut} & y_{ut} & z_{ut} \end{array}\right]}^{T}$ denotes true position of the sea target in the unstable carrier coordinate systematic of the platform, $B_{s}$ denotes the measurement systematic error and $B_{w}$ denotes the zero-mean white Gaussian measurement noise with variance $R_{Bw}=diag(\sigma_{wr}^{2},\sigma_{wb}^{2},\sigma_{we}^{2})$, $h_{rtc}$ denotes transformation from cartesian coordinates to spherical coordinates.

(2) $$h_{rtc}(X)=\left[\begin{array}{c} r\\ b\\ e \end{array}\right]=\left[\begin{array}{c} \sqrt{x^{2}+y^{2}+z^{2}}\\ \arctan(\frac{y}{x})\\ \arctan(\frac{z}{\sqrt{x^{2}+y^{2}}}) \end{array}\right]$$

Let the polar coordinates of $X_{ut}$ be $Z_{ut}={\left[\begin{array}{ccc} r_{ut} & b_{ut} & e_{ut} \end{array}\right]}^{T}$ and the right-angle coordinates of $Z_{um}$ be $X_{um}={\left[\begin{array}{ccc} x_{um} & y_{um} & z_{um} \end{array}\right]}^{T}$, so the measurement equation of the aerial moving platform is rewritten as

(3) $$X_{um}=h_{ctr}(Z_{ut}(k)+B_{s}+B_{w})$$

where $h_{ctr}$ denotes transformation from spherical coordinates to cartesian coordinates.

(4) $$h_{ctr}(Z)=\left[\begin{array}{c} x\\ y\\ z \end{array}\right]=\left[\begin{array}{c} r\cos b\cos e\\ r\sin b\cos e\\ r\sin e \end{array}\right]$$

Since $B_{s}$ and $B_{w}$ is small compared to $Z_{ut}$, Equation (3) can be approximated by a first-order Taylor linear expansion

(5) $$X_{um}\approx h_{ctr}(Z_{ut})+H_{ctr}(Z_{ut})B_{s}+H_{ctr}(Z_{ut})B_{w}$$

where $H_{ctr}(Z_{ut})$ denotes the Jacobi matrix of $h_{ctr}(Z_{ut})$ with respect to $Z_{ut}$.

(6) $$H_{ctr}(Z)=\left[\begin{array}{ccc} \cos b\cos e & -r\sin b\cos e & -r\cos b\sin e\\ \sin b\cos e & r\cos b\cos e & -r\sin b\sin e\\ \sin e & 0 & r\cos e \end{array}\right]$$

### 2.2. Position Alignment

When processing and analyzing the data measured by multiple aerial moving platforms, it is necessary to transform the target position information measured by different platforms into a unified coordinate systematic, called position alignment. In this paper, the sea target position information is standardized to a local geodetic reference system under the fusion center.

According to the sequence from unstable carrier coordinate systematic to stable carrier coordinate systematic to earth coordinate systematic and finally to the local georeferencing systematic under the fusion center, the detection information of the aerial moving platform on the sea target is transformed into the local geodetic reference system under the fusion center based on the high-precision geodetic coordinates of the air movable platform $X_{ep}={\left[\begin{array}{ccc} L_{p} & B_{p} & H_{p} \end{array}\right]}^{T}$, attitude information $\mu ={\left[\begin{array}{ccc} \Psi & \theta & \phi \end{array}\right]}^{T}$, and geodetic coordinates of the fusion center $X_{er}={\left[\begin{array}{ccc} L_{r} & B_{r} & H_{r} \end{array}\right]}^{T}$.

The coordinates in the local geodetic reference system under the fusion center transformed by the aerial moving platform to the sea target measurement data are

(7) $$X_{lrt}=T_{lte}{\left(X_{er}\right)}^{T}T_{lte}(X_{ep})T_{uts}(\mu )X_{lpt}+X_{lrp}$$

where $X_{lpt}$ denotes the coordinates of the sea target under the unstable carrier coordinate systematic centered on the aerial moving platform, $X_{lrp}$ denotes the coordinates of the aerial moving platform under the local geodetic reference system at the fusion center, $T_{lte}(X_{e})$ the transformation of the local geodetic reference system to the Earth coordinate systematic,

(8) $$T_{lte}(X_{e})=\left[\begin{array}{ccc} -\sin L & 0 & \cos L\\ \cos L & 0 & \sin L\\ 0 & 1 & 0 \end{array}\right]\left[\begin{array}{ccc} 1 & 0 & 0\\ 0 & \cos B & \sin B\\ 0 & -\sin B & \cos B \end{array}\right]$$

$T_{uts}(\mu )$ denotes the transformation of the unstable carrier coordinate systematic to the stable carrier coordinate systematic,

(9) $$T_{uts}(\mu )=\left[\begin{array}{ccc} \cos\psi & -\sin\psi & 0\\ \sin\psi & \cos\psi & 0\\ 0 & 0 & 1 \end{array}\right]\left[\begin{array}{ccc} 1 & 0 & 0\\ 0 & \cos\theta & -\sin\theta \\ 0 & \sin\theta & \cos\theta \end{array}\right]\left[\begin{array}{ccc} \cos\phi & 0 & \sin\phi \\ 0 & 1 & 0\\ -\sin\phi & 0 & \cos\phi \end{array}\right]$$

**Figure 1:**
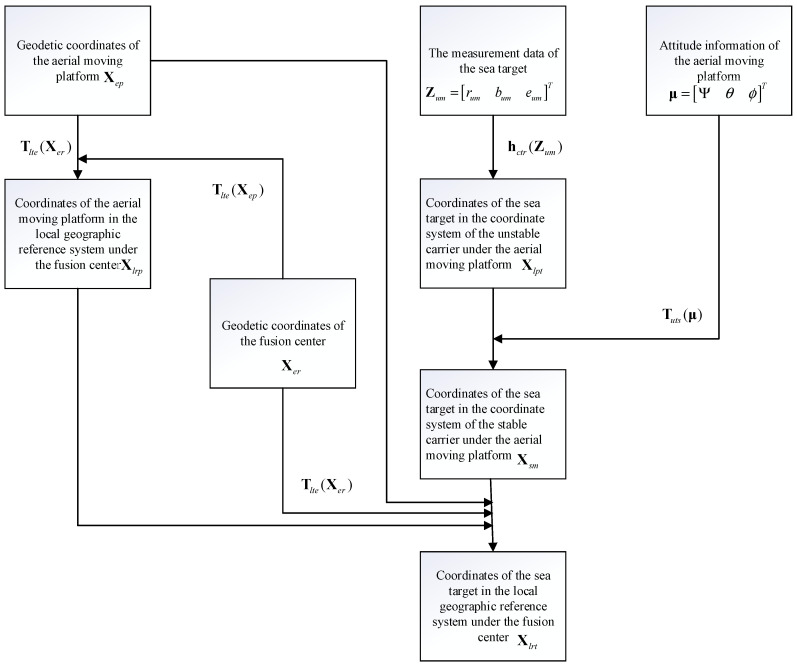
Flowchart of position alignment.

In summary, the flowchart of the transformation of the detection information from the aerial moving platform to the local geodetic reference system under the fusion center is shown in Figure 1.

## 3. New Spatial Registration Algorithm

### 3.1. Observation Error Analysis

Observation errors refer to the differences between the true values of measurements. These errors can occur due to a variety of factors, including calibration drift, electronic noise, or environmental disturbances. Specifically, the observation error of the aerial moving platform to the sea target mainly comes from two parts: sensor measurement error $B_{r}$ and platform attitude angle error $B_{p}$, and both contain systematic and random errors.

(10) $$B=B_{s}+B_{w}$$

(11) $$B_{r}=B_{rs}+B_{rw}$$

(12) $$B_{p}=B_{ps}+B_{pw}$$

where ${Β}_{s}={\left[\begin{array}{ccc} B_{sr} & B_{sb} & B_{se} \end{array}\right]}^{T}$, $B_{rs}={\left[\begin{array}{ccc} B_{rsr} & B_{rsb} & B_{rse} \end{array}\right]}^{T}$, $B_{ps}={\left[\begin{array}{ccc} B_{ps\Psi } & B_{ps\theta } & B_{ps\phi } \end{array}\right]}^{T}$ respectively denote the systematic error of observation error, sensor error, and attitude angle error; $B_{w}={\left[\begin{array}{ccc} B_{wr} & B_{wb} & B_{we} \end{array}\right]}^{T}$, $B_{rw}={\left[\begin{array}{ccc} B_{rwr} & B_{rwb} & B_{rwe} \end{array}\right]}^{T}$, and $B_{pw}={\left[\begin{array}{ccc} B_{pw\Psi } & B_{pw\theta } & B_{pw\phi } \end{array}\right]}^{T}$ respectively denote the random error of observation error, sensor error, and attitude angle error.

Since the data types of sensor error and attitude angle error are different, they cannot be directly fused by adding or subtracting them in a simple way. Typically, sensor error and attitude angle error are treated as separate factors due to their different data types. However, this approach can greatly increase computation and model complexity while also potentially impacting real-time tracking and tracking accuracy. Considering that both sensor error and attitude angle error finally act on the measurement data, the attitude error of the aerial moving platform can be converted into a part of the sensor error by analyzing the effect of attitude angle error on the sensor observation data.

### 3.2. Error Model

The attitude angle of the air platform μ is provided by the platform inertial guidance and other equipment in real-time, and there is a deviation from the true value, which is noted as $B_{p}={\left[\begin{array}{ccc} \Delta \Psi & \Delta \theta & \Delta \phi \end{array}\right]}^{T}$

(13) $$\{\begin{array}{c} \Psi =\Psi_{t}+\Delta \Psi =\Psi_{t}+\Delta \Psi_{s}+\Delta \Psi_{\omega }\\ \theta =\theta_{t}+\Delta \theta =\theta_{t}+\Delta \theta_{s}+\Delta \theta_{\omega }\\ \phi =\phi_{t}+\Delta \phi =\phi_{t}+\Delta \phi_{s}+\Delta \phi_{\omega } \end{array}$$

where $\Psi_{t}$, $\theta_{t}$, $\phi_{t}$ respectively denote the true value of yaw angle, pitch angle and roll angle; $\Delta \Psi_{s}$, $\Delta \theta_{s}$, $\Delta \phi_{s}$ respectively denote the systematic error of yaw angle, pitch angle and roll angle; $\Delta \Psi_{w}$, $\Delta \theta_{w}$, $\Delta \phi_{w}$ respectively denote the random error of yaw angle, pitch angle and roll angle.

In general, the attitude angle deviation of the aerial moving platform is small, and the sea target position is transformed from the carrier stable coordinate systematic to the carrier unstable coordinate systematic according to the attitude angle

(14) $$X_{um}=T_{stu}(\mu )X_{sm}=T^{T}{}_{uts}(\mu )X_{sm}$$

where $X_{sm}={\left[\begin{array}{ccc} x_{sm} & y_{sm} & z_{sm} \end{array}\right]}^{T}$ denotes the coordinate of the measured value of the sea target in the carrier stable coordinate systematic.

By considering the true value of the attitude angle as a function of its measurement and error, Equation (14) can be approximated by a first-order Taylor expansion at the measurement of the attitude angle. The derivation is given in Appendix A.

(15) $$\begin{array}{l} X_{um}\approx (T_{stu}(\mu )-M\Delta \phi -N\Delta \theta -O\Delta \psi )X_{sm}\\ =T_{stu}(\mu )X_{sm}-C_{1}\Delta \phi -C_{2}\Delta \theta -C_{3}\Delta \psi \end{array}$$

where:

$$M=\left[\begin{array}{ccc} m_{11} & m_{12} & m_{13}\\ 0 & 0 & 0\\ m_{31} & m_{32} & m_{33} \end{array}\right]N=\left[\begin{array}{ccc} n_{11} & n_{12} & n_{13}\\ n_{21} & n_{22} & n_{23}\\ n_{31} & n_{32} & n_{33} \end{array}\right]O=\left[\begin{array}{ccc} o_{11} & o_{12} & 0\\ o_{21} & o_{22} & 0\\ o_{31} & o_{32} & 0 \end{array}\right]$$

$$C_{1}=\left[\begin{array}{c} c_{\Delta \phi x}\\ c_{\Delta \phi y}\\ c_{\Delta \phi z} \end{array}\right]C_{2}=\left[\begin{array}{c} c_{\Delta \theta x}\\ c_{\Delta \theta y}\\ c_{\Delta \theta z} \end{array}\right]C_{3}=\left[\begin{array}{c} c_{\Delta \Psi x}\\ c_{\Delta \Psi y}\\ c_{\Delta \Psi z} \end{array}\right]$$

According to Equation (14), the distance measurement of the target by the aerial moving platform will not change during the transformation of the sea target from the stable carrier coordinate systematic to the unstable carrier coordinate systematic. The distance measurement is not affected by the attitude angle, so the sensor distance measurement model is

(16) $$r_{um}=r_{ut}+B_{rsr}+B_{rwr}$$

According to Equation (15), the measured elevation of the sea target in the unstable carrier coordinate systematic considering only the attitude angle error can be obtained:

(17) $$e_{upm}=\arcsin\frac{z_{ut}-c_{\Delta \phi z}\Delta \phi -c_{\Delta \theta z}\Delta \theta -c_{\Delta \Psi z}\Delta \Psi }{r_{um}}$$

Considering that ΔΨ, Δθ, Δϕ is small enough, Equation (17) can be expanded into a first-order Taylor

(18) $$e_{upm}\approx e_{ut}-\frac{c_{\Delta \phi y}}{r_{hpm}}\Delta \phi -\frac{c_{\Delta \theta z}}{r_{hpm}}\Delta \theta -\frac{c_{\Delta \Psi y}}{r_{hpm}}\Delta \Psi$$

where $r_{hpm}=\sqrt{x_{um}^{2}+y_{um}^{2}}$.

In summary, the elevation measurement model is obtained

(19) $$e_{um}=-\frac{c_{\Delta \phi y}}{r_{hpm}}\Delta \phi_{w}-\frac{c_{\Delta \theta z}}{r_{hpm}}\Delta \theta_{w}-\frac{c_{\Delta \Psi y}}{r_{hpm}}\Delta \Psi_{w}$$

In the same way, the sensor azimuth measurement model is obtained

(20) $$b_{um}=-\frac{c_{\Delta \phi x}}{r_{hpm}^{2}}\Delta \phi_{w}-\frac{c_{\Delta \theta x}y_{um}-c_{\Delta \theta y}x_{um}}{r_{hpm}^{2}}\Delta \theta_{w}-\frac{c_{\Delta \Psi x}y_{um}-c_{\Delta \Psi y}x_{um}}{r_{hpm}^{2}}\Delta \Psi_{w}$$

### 3.3. Systematic Error Consistency Matrix

The effect of attitude angle error on the measured data of platform sensors has been obtained, and the connection between sensor error and attitude angle error has been established. The effect of systematic error on the measured values in the measurement equation is obtained from Equations (16), (19) and (20) to construct a systematic error consistency matrix $h_{sp}$ to reduce the systematic error

(21) $$B_{s}=h_{sp}\left[\begin{array}{c} B_{rs}\\ B_{ps} \end{array}\right]$$

where:

$$h_{sp}=\left[\begin{array}{cccccc} 1 & 0 & 0 & 0 & 0 & 0\\ 0 & 1 & 0 & -\frac{c_{\Delta \Psi x}y_{um}-c_{\Delta \Psi y}x_{um}}{r_{hpm}^{2}} & -\frac{c_{\Delta \theta x}y_{um}-c_{\Delta \theta y}x_{um}}{r_{hpm}^{2}} & -\frac{c_{\Delta \phi x}}{r_{hpm}^{2}}\\ 0 & 0 & 1 & -\frac{c_{\Delta \Psi z}}{r_{hpm}} & -\frac{c_{\Delta \theta z}}{r_{hpm}} & -\frac{c_{\Delta \phi z}}{r_{hpm}} \end{array}\right]$$

### 3.4. Sea Target Tracking

The basic framework of the new tracking algorithm for sea targets proposed in this paper is based on the Kalman filtering of IMM. The difference is that a new pseudo-measurement equation is constructed from the measurements of the same sea target by two different aerial moving platforms.

First, we synthesize Equations (5), (7) and (21) to rewrite the measurement equation of the aerial moving platform and divide the measured value into three parts: true position of the sea target, systematic error, and random error

(22) $$\begin{array}{ll} X_{lrt}= & T_{lte}{\left(X_{er}\right)}^{T}T_{lte}(X_{ep})T_{uts}(\mu )h_{ctr}(Z_{t})+X_{lrp}+\\ & T_{lte}{\left(X_{er}\right)}^{T}T_{lte}(X_{ep})T_{uts}(\mu )H_{ctr}(Z_{t})h_{sp}B_{s}+\\ & T_{lte}{\left(X_{er}\right)}^{T}T_{lte}(X_{ep})T_{uts}(\mu )H_{ctr}(Z_{t})B_{w} \end{array}$$

let

(23) $$H_{1}=T_{lte}{\left(X_{er}\right)}^{T}T_{lte}(X_{ep})T_{uts}(\mu )H_{ctr}(Z_{t})$$

(24) $$B_{wn}=T_{lte}{\left(X_{er}\right)}^{T}T_{lte}(X_{ep})T_{uts}(\mu )H_{ctr}(Z_{t})B_{w}$$

Equation (24) can be written

(25) $$X_{lrt}=T_{lte}{\left(X_{er}\right)}^{T}T_{lte}(X_{ep})T_{uts}(\mu )h_{ctr}(Z_{t})+X_{lrp}+H_{1}h_{sp}B_{s}+B_{wn}$$

in which $B_{wn}$ can be approximated as zero-mean Gaussian white noise with a covariance matrix

$$R_{wn}=T_{lte}{\left(X_{er}\right)}^{T}T_{lte}(X_{ep})T_{uts}(\mu )H_{ctr}(Z_{t})R_{Bw}H^{T}{}_{ctr}(Z_{t})T^{T}{}_{uts}(\mu )T^{T}{}_{lte}(X_{ep})T_{lte}(X_{er})$$

In practical operations, the true value of the target measurement data $Z_{t}$ is often difficult to obtain, so the measured value $Z_{m}$ is used instead of $Z_{t}$.

The pseudo-measurement equation is constructed based on the uniqueness of the location of the sea target. From Equation (22), the true position of the sea target in the local geographic reference systematic under the fusion center is

(26) $$HX=T_{lte}{\left(X_{er}\right)}^{T}T_{lte}(X_{ep})T_{uts}(\mu )h_{ctr}(Z_{t})+X_{lrp}$$

where:

$$H=\left[\begin{array}{cccccc} 1 & 0 & 0 & 0 & 0 & 0\\ 0 & 0 & 1 & 0 & 0 & 0\\ 0 & 0 & 0 & 0 & 1 & 0 \end{array}\right]$$

$X={\left[\begin{array}{cccccc} x & v_{x} & y & v_{y} & z & v_{z} \end{array}\right]}^{T}$ is the state of the target under the local geographic reference systematic under the fusion center.

Therefore, the measurement equation of the aerial moving platform is rewritten as

(27) $$X_{lrt}=HX+H_{1}h_{sp}B_{s}+B_{wn}$$

The measurement equations of the aerial moving platforms A and B on the sea target are obtained separately:

(28) $$X_{lrtA}=HX_{A}+H_{1A}h_{\mathrm{spA}}B_{\mathrm{sA}}+B_{\mathrm{wnA}}$$

(29) $$X_{lrtB}=HX_{B}+H_{1B}h_{spB}B_{sB}+B_{wnB}$$

It is easy to obtain according to the consistency of the target location,

(30) $$HX_{A}=HX_{B}$$

Subtracting Equation (28) from Equation (29)

(31) $$\begin{array}{ll} X_{lrtA}-X_{lrtB} & =HX_{A}+H_{1A}h_{spA}B_{sA}+B_{wnA}-HX_{B}-H_{1B}h_{spB}B_{sB}-B_{wnB}\\ & =\left[\begin{array}{cc} H_{1A}h_{spA} & -H_{1B}h_{spB} \end{array}\right]\left[\begin{array}{c} B_{sA}\\ B_{sB} \end{array}\right]+B_{wnA}-B_{wnB} \end{array}$$

From Equation (31), we can obtain the expressions of the sensor systematic error and attitude angle systematic error about the aerial moving platform measurement

(32) $$\left[\begin{array}{c} B_{sA}\\ B_{sB} \end{array}\right]=G^{+}(X_{lrtA}-X_{lrtB}-B_{wnA}+B_{wnB})$$

where: $G^{+}$ is the generalized inverse of $\left[\begin{array}{cc} H_{1A}h_{spA} & -H_{1B}h_{spB} \end{array}\right]$ and its expression is

(33) $$G^{+}={\left[\begin{array}{cc} H_{1A}h_{spA} & -H_{1B}h_{spB} \end{array}\right]}^{T}{\left(\left[\begin{array}{cc} H_{1A}h_{spA} & -H_{1B}h_{spB} \end{array}\right]{\left[\begin{array}{cc} H_{1A}h_{spA} & -H_{1B}h_{spB} \end{array}\right]}^{T}\right)}^{-1}$$

Equation (28) and Equation (29) can be added to obtain

(34) $$\begin{array}{ll} X_{lrtA}+X_{lrtB} & =HX_{A}+H_{1A}h_{\mathrm{spA}}B_{\mathrm{sA}}+B_{\mathrm{wnA}}+HX_{B}+H_{1B}h_{spB}B_{sB}+B_{wnB}\\ & =2HX+\left[\begin{array}{cc} H_{1A}h_{spA} & H_{1B}h_{spB} \end{array}\right]\left[\begin{array}{c} B_{sA}\\ B_{sB} \end{array}\right]+B_{\mathrm{wnA}}+B_{wnB} \end{array}$$

Substitute Equation (32) into Equation (34) to eliminate systematic errors

(35) $$\begin{array}{c} X_{lrtA}+X_{lrtB}=2HX+\\ \left[\begin{array}{cc} H_{1A}h_{spA} & H_{1B}h_{spB} \end{array}\right]G^{+}(X_{lrtA}-X_{lrtB}-B_{wnA}+B_{wnB})+B_{\mathrm{wnA}}+B_{wnB} \end{array}$$

let

(36) $$\begin{array}{c} N=\\ \left[\begin{array}{cc} H_{1A}h_{spA} & H_{1B}h_{spB} \end{array}\right]{\left[\begin{array}{cc} H_{1A}h_{spA} & -H_{1B}h_{spB} \end{array}\right]}^{T}{\left(\left[\begin{array}{cc} H_{1A}h_{spA} & -H_{1B}h_{spB} \end{array}\right]{\left[\begin{array}{cc} H_{1A}h_{spA} & -H_{1B}h_{spB} \end{array}\right]}^{T}\right)}^{-1} \end{array}$$

Substituting into Equation (35) gives

(37) $$(E_{3\times 3}-N)X_{lrtA}+(E_{3\times 3}+N)X_{lrtB}=2HX+(E_{3\times 3}-N)B_{wnA}+(E_{3\times 3}+N)B_{wnB}$$

where: $E_{3\times 3}$ denotes the third-order unit array.

Let

(38) $$z=(E_{3\times 3}-N)X_{lrtA}+(E_{3\times 3}+N)X_{lrtB}$$

(39) $$B_{wf}=(E_{3\times 3}-N)B_{wnA}+(E_{3\times 3}+N)B_{wnB}$$

where the error covariance matrix of $B_{wnA}$ is $R_{wnA}$ and the error covariance matrix of $B_{wnB}$ is $R_{wnB}$.

In summary, Equation (37) can be written as

(40) $$z=2HX+B_{wf}$$

where: $B_{wf}$ approximates zero-mean Gaussian white noise with an error covariance matrix of

$$R_{wf}=(E_{3\times 3}-N)R_{wnA}{\left(E_{3\times 3}-N\right)}^{T}+(E_{3\times 3}+N)R_{wnB}{\left(E_{3\times 3}+N\right)}^{T}$$

**Figure 2:**
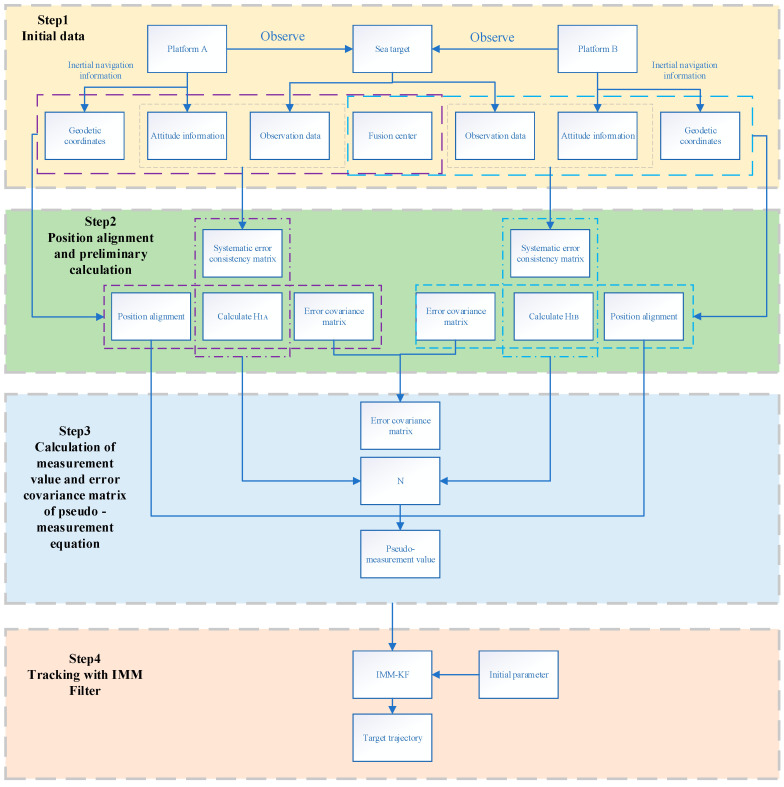
Algorithm flow chart.

The pseudo-measurement equation to reduce the impact of systematic errors in both sensor measurements and platform attitude angles is constructed, and the random sensor error and attitude angle random error is further reduced by combining IMM and Kalman filtering techniques. Now we can complete the fast-tracking of the sea target and the specific process is shown in Figure 2.

## 4. Experimental Verification

### 4.1. Simulation Experiment

To first verify the feasibility of the above algorithm, simulation experiments are conducted and compared with the existing algorithm. Linear Pseudo Linear Kalman Filter (PLKF) and nonlinear Square Root Cubature Kalman Filter (SRCKF) are used to track the target position, respectively, where EC represents the error estimation method in the literature [12], thus combining four comparison methods: IMM-PLKF, IMM-SRCKF, IMM-ECPLKF, and IMM-ECSRCKF.

#### 4.1.1. Experimental Parameter Setting

The fusion center is located at coordinates $X_{er}={\left[\begin{array}{ccc} 114.6354341400^{\circ } & 30.4293397700^{\circ } & 4.6\;m \end{array}\right]}^{T}$. The local geographic coordinate of the aerial moving platforms A and B under the fusion center are $\left[\begin{array}{ccc} 16200\;m & 11700\;m & 1500\;m \end{array}\right]$ and $\left[\begin{array}{ccc} 11700\;m & 16200\;m & 1500\;m \end{array}\right]$. The initial position of the target is located at a reference point with a geodesic length of 198 km and a geodetic azimuth of 60°. Set the data sampling rate f=20 Hz. The standard deviation of the sensor errors (distance, azimuth, elevation) and attitude angle errors (yaw, pitch, roll) of the aerial moving platforms A and B is shown in Table 1 and Table 2. The root mean square error (RMSE) is used as the error evaluation index. The initial value of the tracking of the target is based on the observations of the target.

In this paper, two models are used in the IMM algorithm: Constant Velocity model (CV) and Constant Turning model (CT). $F_{V}$ and $F_{T}$ are the state transfer matrices of CV and CT.

$$F_{V}=\left[\begin{array}{llllll} 1 & 1/f & 0 & 0 & 0 & 0\\ 0 & 1 & 0 & 0 & 0 & 0\\ 0 & 0 & 1 & 1/f & 0 & 0\\ 0 & 0 & 0 & 1 & 0 & 0\\ 0 & 0 & 0 & 0 & 1 & 1/f\\ 0 & 0 & 0 & 0 & 0 & 1 \end{array}\right]$$

$$F_{T}=\left[\begin{array}{llllll} 1 & \frac{\sin(\omega /f)}{\omega } & 0 & 0 & 0 & 0\\ 0 & \cos(\omega /f) & 0 & 0 & 0 & 0\\ 0 & 0 & 1 & \frac{\sin(\omega /f)}{\omega } & 0 & 0\\ 0 & 0 & 0 & \cos(\omega /f) & 0 & 0\\ 0 & 0 & 0 & 0 & 1 & \frac{\sin(\omega /f)}{\omega }\\ 0 & 0 & 0 & 0 & 0 & \cos(\omega /f) \end{array}\right]$$

where: ω denotes the angular speed of the turn.

The state transfer probability matrix of IMM is set to Pi.

$$Pi=\left[\begin{array}{ccc} 0.95 & 0.025 & 0.025\\ 0.025 & 0.95 & 0.025\\ 0.025 & 0.025 & 0.95 \end{array}\right]$$

In order to fully verify the feasibility, robustness, and superiority of the algorithm compared with other algorithms, two groups of different simulation experimental scenarios are set up, mainly changing the error settings, in which the simulation duration is all 400 s, and the target motion is uniform linear motion from 0 to 80 s, uniform right-turn motion from 80 to 160 s, uniform linear motion from 160 to 240 s, uniform linear motion from 240 to 320 s, and 320~400 s. The error settings for different scenarios are as follows.

Simulation scenario one: the error settings are the same as in Table 1 and Table 2.

To verify that the newly proposed algorithm is robust and applicable under different systematic error variations, scenario two is designed.

Simulation scenario two: Compared with simulation scenario one, the error of the sensor observation system of the aerial motion platform A changes abruptly at 50–70 s and 240–260 s, and its angular deviation becomes eight times the original one.

#### 4.1.2. Simulation Experiment Result

**Figure 3:**
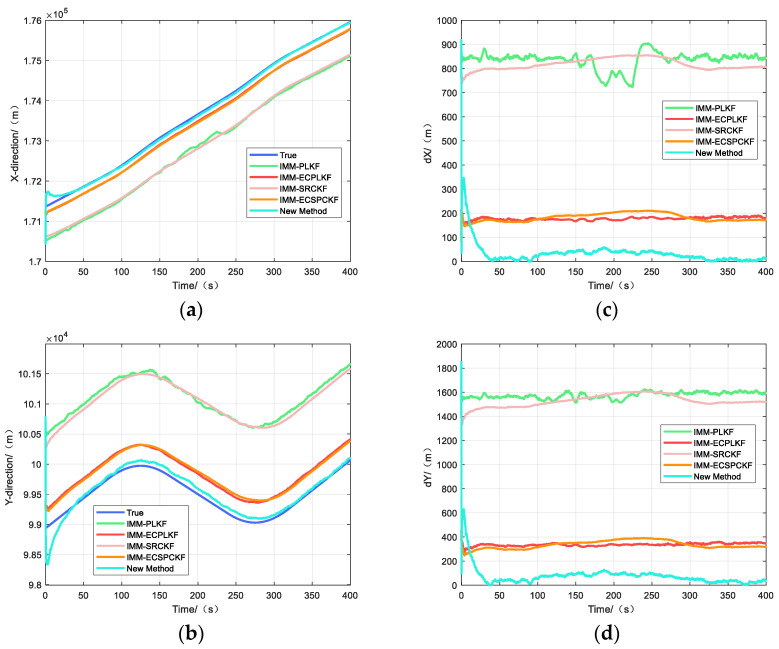
Tracking effect of scene one: (**a**) Tracking effect in x-direction; (**b**) Tracking effect in y-direction; (**c**) Tracking error in x-direction; (**d**) Tracking error in y-direction.

**Figure 4:**
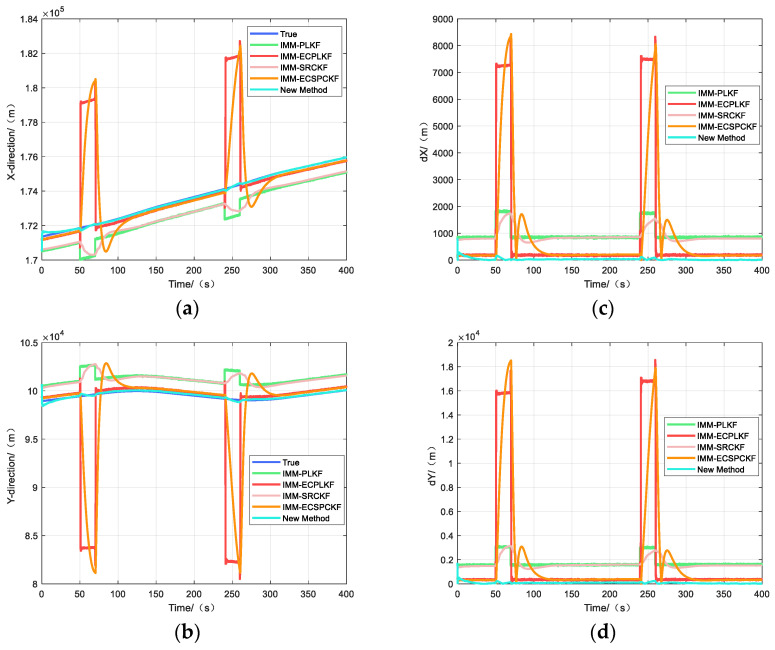
Tracking effect of scene two: (**a**) Tracking effect in x-direction; (**b**) Tracking effect in y-direction; (**c**) Tracking error in x-direction; (**d**) Tracking error in y-direction.

Figure 3 and Figure 4 show the target position estimation accuracy of scenarios one and two, respectively, where the left side indicates the position estimation effects in each direction and the right side indicates the position estimation errors in each direction.

In order to further analyze the causes of IMM-PLKF and IMM-PLKF tracking errors, we carried out simulation experiments by adding random sensor error, sensor systematic error, and attitude error, respectively, on the basis of scenario 1 target motion. The tracking results are shown in Table 3.

As can be seen from Figure 3 and Table 4, the tracking accuracy of the new algorithm is better than IMM-ECPLKF and IMM-ECSRCKF in scenario one, and IMM-PLKF and IMM-SRCKF have the worst tracking effect. In scenario one, the tracking accuracy of the new algorithm is improved by about three times compared with IMM-ECPLKF and IMM-ECSRCKF. IMM-PLKF and IMM-SRCKF can only eliminate most of the random errors of the sensors, and the bias caused by the systematic errors of the sensor observation and the systematic errors of the attitude angle is difficult to eliminate. When the value of the systematic errors is large, it will have a greater impact on the estimation accuracy of the target tracking. IMM-ECPLKF and IMM-ECSRCKF can eliminate most of the sensor observation system errors and attitude angle system errors, but the accuracy of the elimination depends on the model priori information of the sensor and inertial guidance system errors, and when the uncertainty is too large, the tracking accuracy of IMM-ECPLKF and IMM-ECSRCKF will be reduced, sometimes even inferior to that of IMM-PLKF and IMM-ECSRCKF, resulting in filter divergence. The new algorithm solves this problem well by skipping the process of calculating the systematic error mathematically and tracking the target directly. This can get a higher tracking accuracy, but in the initial stage of tracking, the starting tracking error is larger due to using the target observation as the initial value of tracking, and the convergence speed is slightly slower than other algorithms.

Figure 4 shows the tracking effect when the system error changes abruptly, when the system error is no longer constant, or when it changes slowly. The tracking errors of IMM-PLKF and IMM-SRCKF increase accordingly when there is an abrupt change in the systematic error. However, the errors of IMM-ECPLKF and IMM-ECSRCKF are significantly higher than those of IMM-PLKF and IMM-SRCKF during abrupt changes. The reason is that the error estimation method proposed in the literature [12] is essentially a first-order expansion of the error characteristics, similar to the EKF estimation, which has no better estimation effect on such abrupt system errors when the nonlinearity of the system error characteristics is too large and even causes the error estimation results to scatter during the abrupt change because of the large abrupt change errors. In contrast, the new algorithm shows better estimation accuracy and suppression effects on time-varying systematic errors, resulting in less influence from abrupt errors on its tracking performance. Furthermore, Table 5 demonstrates that the nonlinear filter SRCKF outperforms the linear filter PLKF slightly in terms of tracking performance.

**Figure 5:**
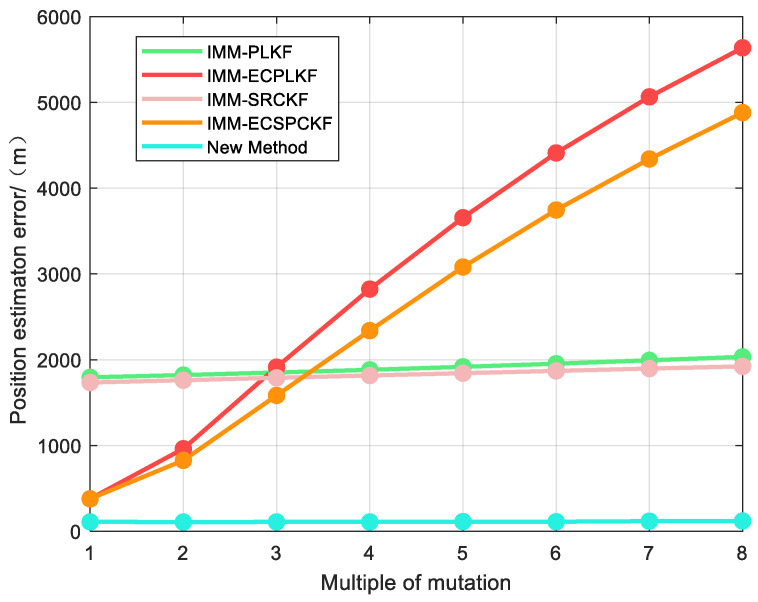
Position estimation errors under different mutation conditions.

To further evaluate the robustness of the new algorithm against error effects, we conducted experiments by gradually increasing the error mutation multiplier from 1 to 8. The results are as follows: Figure 5 and Table 6 illustrate the tracking errors of different algorithms under varying error mutation multipliers.

From Figure 5 and Table 6, we can observe that the new method consistently achieves the highest tracking accuracy across various mutation conditions. The tracking error experiences a nominal change of approximately 10 m when the mutation multiplier increases from 1 to 8. This implies that the tracking effect is almost unaffected by changes in the mutation multiplier, indicating that the new method exhibits superior robustness. One explanation for the poor performance of IMM-ECPLKF and IMM-ECSRCKF is that the error estimation model may not be suitable for cases with large error variations, leading to poor spatial registration results and increased tracking errors.

It is worth noting that the IMM-PLKF and IMM-SRCKF exhibit slower changes as the mutation multiplier increases since the mutation time is shorter. As a result, these methods demonstrate smaller changes in total tracking error compared to the IMM-ECPLKF and IMM-ECSRCKF. Table 6 illustrates that the tracking error increases by approximately 200 m as the mutation multiplier increases from 1 to 8, indicating an increasing trend in tracking error with the mutation multiplier.

Table 7 displays the average single-step time of each algorithm during the simulation. The results indicate that the PLKF algorithm is faster compared to the SRCKF algorithm, primarily due to the additional calculation required by the SRCKF for sampling points, which increases the operation time. However, IMM-PLKF and IMM-SRCKF demonstrate better tracking accuracy than the PLKF and SRCKF, as the error compensation algorithm requires an initial error estimation step.

In contrast, the new algorithm has the shortest single-step time among all methods, making it computationally efficient. This is primarily due to the fact that the new algorithm does not require individual error estimation or filtering of individual platforms. As a result, it greatly improves computational efficiency.

### 4.2. Practical Experiment

To further verify the effectiveness of the algorithm, we conducted an on-lake experiment in a water area in Ezhou City, Hubei Province.

#### 4.2.1. Experimental Equipment

This experimental system consists of an information processor (a three-screen tablet computer), YAR28 (N) radar, Ku02 radar, combined navigation equipment, RTK, etc. Several major experimental pieces of equipment are briefly introduced below. Due to the difficulty of carrying the existing radar sensors on the UAV, two radars combined with inertial navigation equipment with errors were used to simulate the aerial moving platforms for observation.

**Figure 6:**
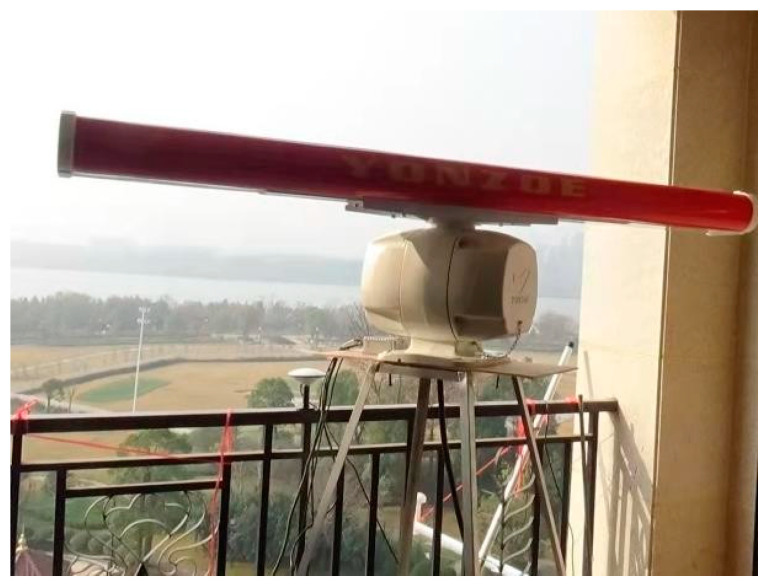
YAR28(N) Radar.

The YAR28(N) radar is a continuous wave systematic sea surveillance network radar with a strong ability to detect small targets, good suppression of sea clutter, and superior target tracking performance. The appearance of this radar is shown in Figure 6, and the main parameters are shown in Table 8.

**Figure 7:**
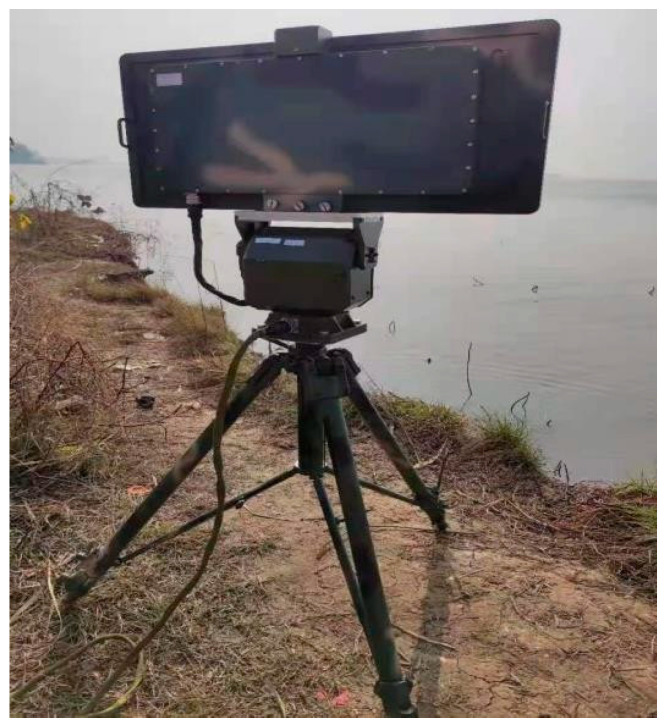
Ku02 radar.

Ku02 radar is a pulse systematic radar that can effectively track low and small slow targets. The appearance of this radar is shown in Figure 7, and the main parameters are shown in Table 9.

**Figure 8:**
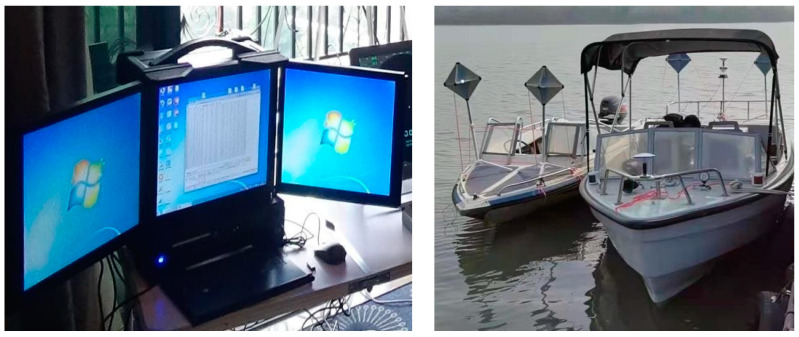
Information processor and target.

The information processor uses a three-screen tablet computer, and the target uses a small boat, as shown in Figure 8.

#### 4.2.2. Experimental Procedure

**Figure 9:**
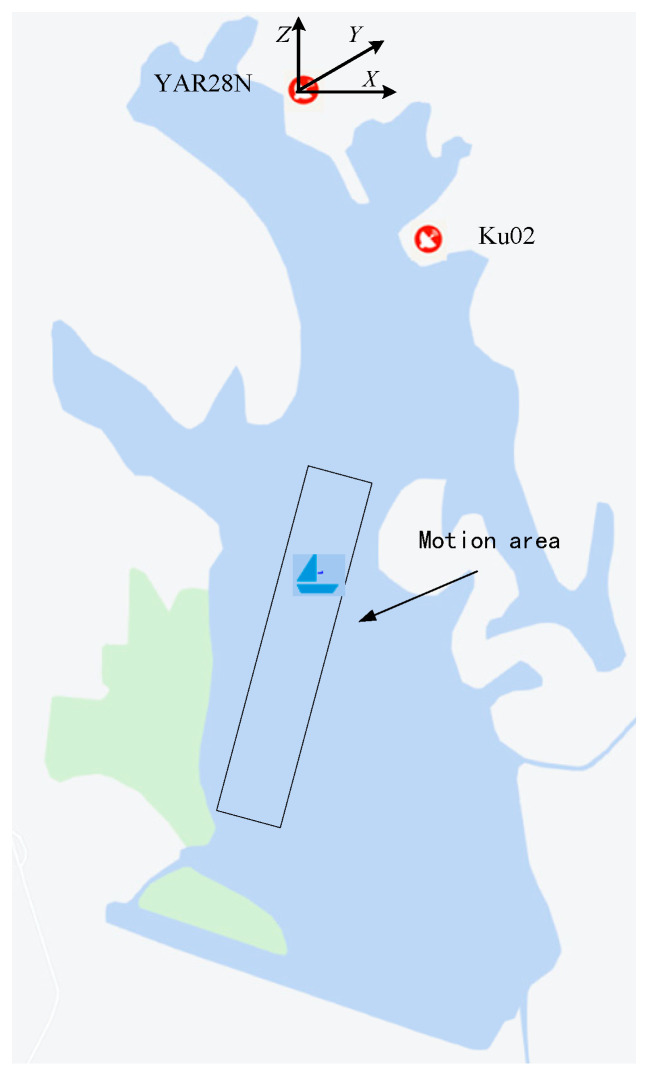
Experimental situation.

In the experiment, two small boats were used as targets to make maneuvering movements in the designated waters. The two radars are placed at a fixed location on the shore to simulate two aerial moving platforms for cooperative observation of the target. In order to obtain a better target state fusion estimation effect, a certain angle is formed between the two radars and the target so that the two radars can complete the observation of the target from different angles. The experimental situation is shown in Figure 9, in which the XYZ axis is defined as the northeast sky coordinate system, the X axis is to the east, the Y axis is to the north, and the Z axis points to the zenith.

**Figure 10:**
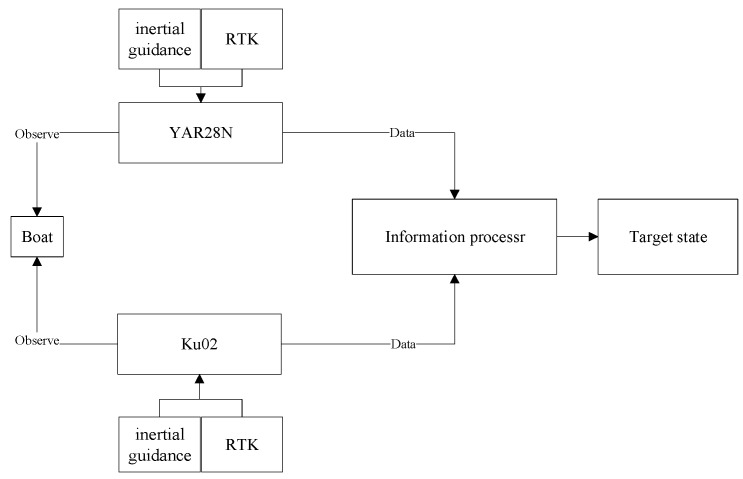
Experimental procedure.

The specific experimental procedure is that the boat navigates in the designated waters and makes maneuvering movements. Then, the two radars are turned on simultaneously, and the two radars search and steadily track the target in real-time to obtain the target’s distance, bearing, and pitch angle information. The inertial guidance equipment outputs the inertial guidance information of the radar with errors, and the RTK equipment outputs the high-precision geodetic coordinates of the radar position. The target observation information obtained by the two radars, as well as the inertial guidance information and the radar geodetic coordinates information, is transmitted to the information processor to finally obtain the state estimation of the target. Experimental procedure is shown in Figure 10.

#### 4.2.3. Experimental Result

**Figure 11:**
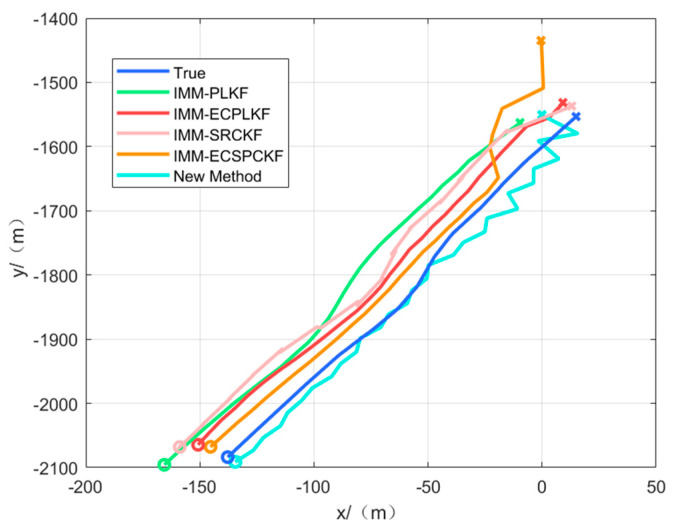
Tracking effect of scene three.

**Figure 12:**
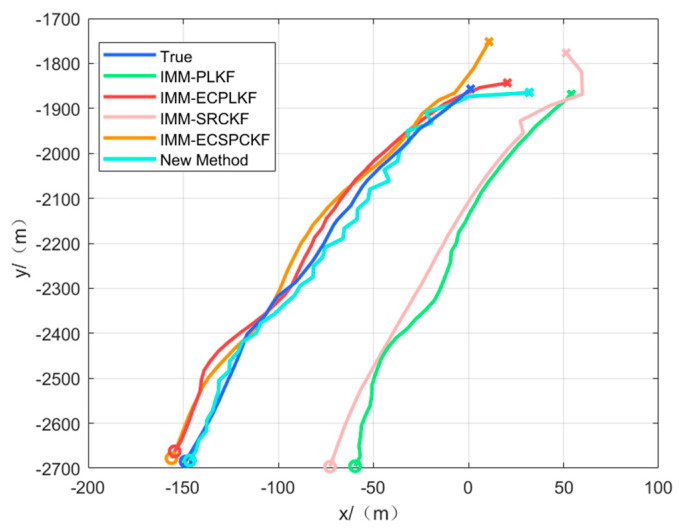
Tracking effect of scenario four.

The algorithm initializes the target tracking with the target’s observations. In order to verify the effectiveness of the proposed algorithm, data collected during on-lake experiments were utilized. Scenarios three and four were executed to demonstrate the algorithm’s ability to track targets along different boat trajectories. The actual tracking results for scenarios three and four are illustrated in Figure 11 and Figure 12, respectively.

When the data processing is performed in the experiment on the lake, the amount of data that can be practically applied after data alignment and wild value elimination is very small. The endpoint and trajectory position estimation errors are calculated for each algorithm. The position estimation errors of scenarios three and four are shown in Table 10 and Table 11.

Based on Figure 11 and Figure 12, we can conclude that the new algorithm exhibits superior tracking performance compared to the IMM-ECPLKF and IMM-ECSRCKF algorithms. Furthermore, the IMM-ECPLKF and IMM-ECSRCKF algorithms demonstrate better tracking ability than the IMM-PLKF and IMM-SRCKF algorithms. During the experiments on the lake, because the environment is relatively stable, the sensor measurement system error and attitude angle system error may not change abruptly, but the error must not be a time-invariant error. The performance of the traditional filter fusion tracking algorithm with error compensation is not as effective as the new algorithm, and because the error characteristics of the system error are not known, a lot of debugging work is required to estimate the system error, which is not conducive to practical engineering applications.

The results of the experiments on the lake further verify the feasibility of the new algorithm and show its superior performance in engineering applications.

## 5. Conclusions

Our study proposes a novel tracking algorithm for sea targets based on the spatial registration of the aerial moving platform sensors. In this study, we design the systematic error consistency matrix to reduce the systematic error and establish the pseudo-measurement equation through the observation of targets by two aerial moving platforms. The novel algorithm aims to achieve more accurate tracking of sea targets. The simulation and on-lake experiments show that the new method has better spatial registration performance than the previous filtering algorithm. When the systematic error is time-varying, the new algorithm can implement stable tracking of sea targets compared to the previous filtering algorithm, which means it has a certain application value in practical scenarios.

## Figures and Tables

**Table 1:** Error characteristics of the aerial motion platform A.

	Standard Deviation of Systematic Error	Standard Deviation of Random Error
Sensor error	$\left(\begin{array}{ccc} 10\;m & 0.3^{\circ } & 0.2^{\circ } \end{array}\right)$	$\left(\begin{array}{ccc} 5\;m & 0.01^{\circ } & 0.01^{\circ } \end{array}\right)$
Attitude angle error	$\left(\begin{array}{ccc} 0.3^{\circ } & 0.3^{\circ } & 0.3^{\circ } \end{array}\right)$	$\left(\begin{array}{ccc} 0.01^{\circ } & 0.01^{\circ } & 0.01^{\circ } \end{array}\right)$

**Table 2:** Error characteristics of the aerial motion platform B.

	Standard Deviation of Systematic Error	Standard Deviation of Random Error
Sensor error	$\left(\begin{array}{ccc} 10\;m & 0.2^{\circ } & 0.2^{\circ } \end{array}\right)$	$\left(\begin{array}{ccc} 5\;m & 0.01^{\circ } & 0.01^{\circ } \end{array}\right)$
Attitude angle error	$\left(\begin{array}{ccc} 0.3^{\circ } & 0.3^{\circ } & 0.3^{\circ } \end{array}\right)$	$\left(\begin{array}{ccc} 0.01^{\circ } & 0.01^{\circ } & 0.01^{\circ } \end{array}\right)$

**Table 3:** Tracking error under different error.

	IMM-PLKF	IMM-SRCKF	New Method
Sensor random error	4.2857	34.3077	40.0531
Sensor systematic error	388.1074	389.3228	57.0316
Attitude error	1408.7420	1367.6781	97.5527
Sensor error and attitude error	1783.8209	1734.1934	126.2673

**Table 4:** Position estimation error of scene one.

Case	Direction	IMM-PLKF	IMM-ECPLKF	IMM-PLKF	IMM-ECSRCKF	New Method
1	X (m)	836.1992	177.7587	818.7084	183.5311	57.7602
	Y (m)	1575.6864	336.4807	1528.7719	336.9392	112.2818
	R (m)	1783.8209	380.5488	1734.1934	383.6818	126.2673

**Table 5:** Position estimation error of scenario two.

Case	Direction	IMM-PLKF	IMM-ECPLKF	IMM-SRCKF	IMM-ECSRCKF	New Method
2	X (m)	981.5877	2323.3987	914.2680	2042.2660	58.5513
	Y (m)	1780.7090	5132.6543	1690.0992	4424.6566	115.5644
	R (m)	2033.3320	5634.0325	1921.5414	4873.2368	129.5507

**Table 6:** Position estimation errors under different mutation conditions.

Multiple	IMM-PLKF	IMM-ECPLKF	IMM-SRCKF	IMM-ECSRCKF	New Method
1	1794.5638	378.3700	1734.3233	378.9424	109.6330
2	1821.3784	962.5777	1761.3032	826.7617	108.0558
3	1851.0328	1913.9942	1789.3128	1583.9521	109.0667
4	1882.4963	2823.5281	1816.3969	2339.2170	108.9363
5	1917.1685	3656.1993	1843.2672	3080.6594	112.8530
6	1953.8306	4410.7501	1869.7388	3745.6466	111.2993
7	1993.3880	5064.5871	1897.0456	4340.3273	117.9363
8	2033.2177	5636.2106	1922.2745	4880.5005	120.1390

**Table 7:** Comparison of mean computational cost.

Algorithm	IMM-PLKF	IMM-ECPLKF	IMM-SRCKF	IMM-ECSRCKF	New Method
Mean computational cost (ms)	0.9578	0.9963	1.1517	1.3420	0.8216

**Table 8:** Main parameters of YAR28(N) radar.

Performance index	Parameter
Radar operating frequency band	X-band
Horizontal beamwidth	1.85°
Vertical Beamwidth	22°
Rotational Speed	24 RPM
Distance accuracy	1% range or 30 m, whichever is greater
Azimuth accuracy	≤1°
Distance of action	0.125~128 NM range

**Table 9:** Main parameters of Ku02 radar.

Performance index	Parameter
Radar operating frequency band	Ku-band
Minimum detection distance	≤150 m
Speed measurement range	0.5~20 m/s
Turntable speed	10°/s~60°/s
Distance Resolution	≤2 m
Azimuth Resolution	≤2°
Distance measurement accuracy	≤0.3 m (within 300 m)
Azimuth accuracy	≤1°

**Table 10:** Endpoint position estimation error of scenarios three and four.

Case	IMM-PLKF	IMM-ECPLKF	IMM-SRCKF	IMM-ECSRCKF	New Method
3	30.2008	23.1548	26.2849	17.7113	8.0571
4	89.7884	23.2870	76.7816	10.2871	2.9354

**Table 11:** Trajectory position estimation error of scenarios three and four.

Case	IMM-PLKF	IMM-ECPLKF	IMM-SRCKF	IMM-ECSRCKF	New Method
3	12.2925	11.9090	11.1139	13.8311	6.6214
4	21.3709	11.6246	20.4188	11.6301	6.6334

## Data Availability

Not applicable.

## Appendix A

(A1)
$$T_{\psi }(\psi )=\left[\begin{array}{ccc} \cos\psi & \sin\psi & 0\\ -\sin\psi & \cos\psi & 0\\ 0 & 0 & 1 \end{array}\right]$$

(A2)
$$T_{\theta }(\theta )=\left[\begin{array}{ccc} 1 & 0 & 0\\ 0 & \cos\theta & \sin\theta \\ 0 & -\sin\theta & \cos\theta \end{array}\right]$$

(A3)
$$T_{\phi }(\phi )=\left[\begin{array}{ccc} \cos\phi & 0 & -\sin\phi \\ 0 & 1 & 0\\ \sin\phi & 0 & \cos\phi \end{array}\right]$$

(A4)
$$\begin{array}{ll} X_{um}\approx & [T_{\phi }(\phi )T_{\theta }(\theta )T_{\psi }(\psi )+\frac{\partial T_{\phi }(\phi )}{\partial \phi }(\phi_{t}-\phi )T_{\theta }(\theta )T_{\psi }(\psi )+\\ & T_{\phi }(\phi )\frac{\partial T_{\theta }(\theta )}{\partial \theta }(\theta_{t}-\theta )T_{\psi }(\psi )+T_{\phi }(\phi )T_{\theta }(\theta )\frac{\partial T_{\psi }(\psi )}{\partial \psi }(\psi_{t}-\psi )]X_{sm} \end{array}$$

Substituting Equation (13) into Equation (A4)

(A5) $$\begin{array}{ll} X_{um}\approx & [T_{\phi }(\phi )T_{\theta }(\theta )T_{\psi }(\psi )-\frac{\partial T_{\phi }(\phi )}{\partial \phi }△\phi T_{\theta }(\theta )T_{\psi }(\psi )-\\ & T_{\phi }(\phi )\frac{\partial T_{\theta }(\theta )}{\partial \theta }\Delta \theta T_{\psi }(\psi )-T_{\phi }(\phi )T_{\theta }(\theta )\frac{\partial T_{\psi }(\psi )}{\partial \psi }△\psi ]X_{sm} \end{array}$$

where:

(A6) $$\frac{\partial {Τ}_{\phi }(\phi )}{\partial \phi }=\left[\begin{array}{ccc} -\sin\phi & 0 & -\cos\phi \\ 0 & 0 & 0\\ \cos\phi & 0 & -\sin\phi \end{array}\right]$$

(A7) $$\frac{\partial T_{\theta }(\theta )}{\partial \theta }=\left[\begin{array}{ccc} 0 & 0 & 0\\ 0 & -\sin\theta & \cos\theta \\ 0 & -\cos\theta & -\sin\theta \end{array}\right]$$

(A8) $$\frac{\partial T_{\psi }(\psi )}{\partial \psi }=\left[\begin{array}{ccc} -\sin\psi & \cos\psi & 0\\ -\cos\psi & -\sin\psi & 0\\ 0 & 0 & 0 \end{array}\right]$$

Integrated Equations (A6)–(A8) to Equation (A5)

(A9) $$\begin{array}{ll} X_{um} & \approx (T_{stu}(\mu )-M\Delta \phi -N\Delta \theta -O\Delta \psi )X_{sm}\\ & =T_{stu}(\mu )X_{sm}-C_{1}\Delta \phi -C_{2}\Delta \theta -C_{3}\Delta \psi \end{array}$$

where:

$$M=\left[\begin{array}{ccc} m_{11} & m_{12} & m_{13}\\ 0 & 0 & 0\\ m_{31} & m_{32} & m_{33} \end{array}\right]=\left[\begin{array}{ccc} -\sin\phi \cos\Psi -\cos\phi \sin\theta \sin\Psi & -\sin\phi \sin\Psi +\cos\phi \sin\theta \cos\Psi & -\cos\phi \cos\theta \\ 0 & 0 & 0\\ \cos\phi \cos\Psi -\sin\phi \sin\theta \sin\Psi & \cos\phi \sin\Psi +\sin\phi \sin\theta \cos\Psi & -\sin\phi \cos\theta \end{array}\right]$$

$$O=\left[\begin{array}{ccc} o_{11} & o_{12} & 0\\ o_{21} & o_{22} & 0\\ o_{31} & o_{32} & 0 \end{array}\right]=\left[\begin{array}{ccc} -\cos\phi \sin\Psi -\sin\phi \sin\theta \cos\Psi & \cos\phi \sin\Psi -\sin\phi \sin\theta \sin\Psi & 0\\ -\cos\phi \cos\Psi & -\cos\theta \sin\Psi & 0\\ -\sin\phi \sin\Psi +\cos\phi \sin\theta \cos\Psi & \sin\phi \cos\Psi +\cos\phi \sin\theta \sin\Psi & 0 \end{array}\right]$$

$$N=\left[\begin{array}{ccc} n_{11} & n_{12} & n_{13}\\ n_{21} & n_{22} & n_{23}\\ n_{31} & n_{32} & n_{33} \end{array}\right]=\left[\begin{array}{ccc} -\sin\phi \cos\theta \sin\Psi & \sin\phi \cos\theta \cos\Psi & \sin\phi \sin\theta \\ \sin\theta \sin\Psi & -\sin\theta \cos\Psi & \cos\theta \\ \cos\phi \cos\theta \sin\Psi & -\cos\phi \cos\theta \cos\Psi & -\cos\phi \sin\theta \end{array}\right]$$

$$C_{1}=\left[\begin{array}{c} c_{\Delta \phi x}\\ c_{\Delta \phi y}\\ c_{\Delta \phi z} \end{array}\right]=\left[\begin{array}{c} -(\sin\phi \cos\Psi +\cos\phi \sin\theta \sin\Psi )x_{sm}-(\sin\phi \sin\Psi -\cos\phi \sin\theta \cos\Psi )y_{sm}-\cos\phi \cos\theta z_{sm}\\ 0\\ (\cos\phi \cos\Psi -\sin\phi \sin\theta \sin\Psi )x_{sm}+(\cos\phi \sin\Psi +\sin\phi \sin\theta \cos\Psi )y_{sm}-\sin\phi \cos\theta z_{sm} \end{array}\right]$$

$$C_{2}=\left[\begin{array}{c} c_{\Delta \theta x}\\ c_{\Delta \theta y}\\ c_{\Delta \theta z} \end{array}\right]=\left[\begin{array}{c} -\sin\phi \cos\theta \sin\Psi x_{sm}+\sin\phi \cos\theta \cos\Psi y_{sm}+\sin\phi \sin\theta z_{sm}\\ \sin\theta \sin\Psi x_{sm}-\sin\theta \cos\Psi y_{sm}+\cos\theta z_{sm}\\ \cos\phi \cos\theta \sin\Psi x_{sm}-\cos\phi \cos\theta \cos\Psi y_{sm}-\cos\phi \sin\theta z_{sm} \end{array}\right]$$

$$C_{3}=\left[\begin{array}{c} c_{\Delta \Psi x}\\ c_{\Delta \Psi y}\\ c_{\Delta \Psi z} \end{array}\right]=\left[\begin{array}{c} -(\cos\phi \sin\Psi +\sin\phi \sin\theta \cos\Psi )x_{sm}+(\cos\phi \sin\Psi -\sin\phi \sin\theta \sin\Psi )y_{sm}\\ -\cos\phi \cos\Psi x_{sm}-\cos\theta \sin\Psi y_{sm}\\ (\cos\phi \sin\theta \cos\Psi -\sin\phi \sin\Psi )x_{sm}+(\sin\phi \cos\Psi +\cos\phi \sin\theta \sin\Psi )y_{sm} \end{array}\right]$$
